# Biomechanical Simulation of Orthodontic En-Bloc Retraction Comparing Compound Technique and Sliding Mechanics Using a HOSEA Robotic Device

**DOI:** 10.3390/bioengineering11020153

**Published:** 2024-02-02

**Authors:** Hisham Sabbagh, Ellen Haas, Uwe Baumert, Corinna Lesley Seidel, Linus Hötzel, Andrea Wichelhaus

**Affiliations:** Department of Orthodontics and Dentofacial Orthopedics, LMU University Hospital, LMU Munich, Goethestrasse 70, 80336 Munich, Germany; ellen.haas@med.uni-muenchen.de (E.H.); uwe.baumert@med.uni-muenchen.de (U.B.); corinna.seidel@med.uni-muenchen.de (C.L.S.); linus.hoetzel@med.uni-muenchen.de (L.H.); kfo.sekretariat@med.uni-muenchen.de (A.W.)

**Keywords:** orthodontics, biomechanics, 3D measurement, force-control, hexapod, retraction, robotics

## Abstract

En-bloc retraction is a common procedure in orthodontic therapy. The application of palatal root torque moments is required to control incisor inclination during retraction, yet studies comparing forces and moments with respect to different mechanics are lacking. This study aimed to investigate the forces and moments during orthodontic en-bloc retraction using a robotic biomechanical simulation system, comparing two distinct approaches: (I) compound technique [stainless steel (SS) combined with nickel-titanium (NiTi)] using industrially pretorqued retraction-torque-archwires (RTA) in combination with NiTi closed coil springs; (II) conventional sliding mechanics using SS archwires with manually applied anterior twist bends in combination with elastic chains. Two dimensions (0.017” × 0.025” and 0.018” × 0.025”) and ten archwires per group were investigated using 0.022” slot self-ligating brackets. Kruskal–Wallis tests with a significance level of α = 0.05 were conducted. The biomechanical simulation showed that en-bloc retraction was characterized by a series of tipping and uprighting movements, differing significantly regarding the examined mechanics. Collateral forces and moments occurred in all groups. Notably, RTA exhibited fewer extrusive forces. The most bodily movement was achieved with the compound technique and the 0.018” × 0.025” RTA. Sliding mechanics exhibited maximum palatal root torque moments of more than 20 Nmm, exceeding recommended values.

## 1. Introduction

Orthodontic space closure is a frequent treatment task, particularly, but not limited to, extraction therapy. Fixed orthodontic appliances are therapeutically most effective in controlling tooth angulation and performing translational tooth movements during space closure [1,2,3]. While small spaces can be effectively closed using sliding mechanics, the closure of larger spaces may require other techniques, such as modified sliding mechanics, frictionless mechanics or the compound technique [4,5,6,7].

When closing larger gaps, e.g., after premolar extractions, teeth are often moved in groups to increase treatment efficiency and control anchorage [8,9]. In the upper jaw, two different procedures are commonly distinguished, in particular: en-masse and en-bloc retraction.

In en-masse retraction, the entire anterior segment, from canine to canine (3-3), is retracted, resulting in a one-step procedure. In contrast, en-bloc retraction is divided into two treatment phases. First, the canine is retracted and subsequently, the anterior segment from the lateral incisor to the lateral incisor (2-2) is retracted [10].

Although en-bloc retraction is criticized in terms of duration [11] and aesthetics because of the temporary gaps it generates [12], it offers advantages in cases with midline discrepancies, crowded anterior teeth, extruded or high canines or flared incisors [13]. In addition, en-bloc retraction requires less anchorage since fewer teeth are moved at the same time [4].

En-bloc retraction is a biomechanically complex procedure, especially during the second phase, when four incisors are to be moved simultaneously in the posterior direction while controlling their labio-lingual inclination. For bodily movement of the anterior segment, the sum vector of the applied forces must pass through the approximate center of resistance of the group of teeth, corresponding to a moment-to-force (M/F) ratio of approximately 10:1 at the center of force [5,6,14]. To obtain the required torsional moment to meet this M/F ratio, in addition to a retractive element such as a closing loop, elastic chains or nickel-titanium (Ni-Ti) coil springs, the use of twist bends or pretorqued retraction archwires has been proposed [5,14].

In view of these considerations and the various components and materials available, several distinct techniques were proposed for en-bloc retraction [15]. In clinical practice, conventional sliding mechanics, i.e., steel archwires in combination with elastic chains, are most frequently used for space closure and en-bloc retraction. The advantages of sliding mechanics are their simplicity and fast application. The disadvantage is the torsional play between bracket and archwire in the straight-wire technique, which necessitates anterior twist bends to maintain the incisor inclination during retraction. The use of sliding mechanics and twist bends can easily lead to periodontal overactivation due to the high Young’s modulus of SS archwires, their low activation range and the difficulty of precise manual bending.

The compound technique was introduced to overcome these challenges and therefore combines SS with NiTi components. The retraction-torque-archwire (RTA), which is also known as torque-segmented arch (TSA), consists of two posterior parts composed of stainless steel to use the advantages of the bending and sliding properties and an anterior pretorqued NiTi segment [16]. The NiTi element has the benefits of superelasticity and enhanced transmission of the preset torque to anticipate possible torque losses resulting from bracket/archwire play.

Although knowledge of the force-moment systems is essential for efficient tooth movement and the avoidance of adverse effects such as hyalinization, pain and root resorption, there is scant evidence regarding these distinct techniques [17,18,19].

The determination of acting forces and moments during complex orthodontic procedures like en-bloc retraction is not possible in vivo. Hence, numerical methods like finite element analysis (FEA) and biomechanical test stands have been established in orthodontic science for this purpose [20,21,22,23]. Robotic force-controlled biomechanical test stands allow the investigation of the behavior of actual orthodontic appliances without the influence of subjectively determined parameters or measurement points [24]. HOSEA is a novel biomechanical test system based on a hexapod platform with parallel kinematics that is autonomously moved by a force-controlled algorithm designed for the investigation of complex multiaxial motion sequences in orthodontics [25]. The algorithm processes the measured forces and moments of the applied mechanics and calculates the resulting movements as a function of feedback parameters.

Aim of this study was to investigate the dynamic course of forces and moments during the simulated second step of en-bloc retraction with different fixed orthodontic mechanics using the robotic HOSEA test device. The null hypothesis states that no significant differences exist in forces and moments between the compound technique (RTA with NiTi-coil springs) and sliding mechanics (SS with elastic chains), across archwire dimensions of 0.017” × 0.025” and 0.018” × 0.025”, in orthodontic en-bloc retraction.

## 2. Materials and Methods

### 2.1. Development of the Experimental Model for En-Bloc Retraction

**Figure 1:**
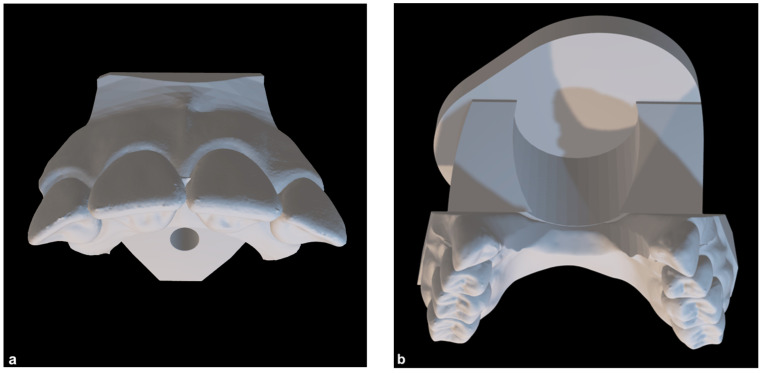
Virtual representation of the experimental maxillary model. (**a**) Anterior segment of teeth; (**b**) posterior segment of teeth with an additionally designed base part.

For the investigation of orthodontic en-bloc retraction, an established biomechanical test stand, HOSEA, was applied with a modified maxillary model [25]. The maxillary model was virtually prepared using the software OnyxCeph^3TM^ (Version 3.2.185; Image Instruments GmbH, Chemnitz, Germany). The first premolars were digitally removed, and the canines were relocated in the extraction space of the first premolars. As a result, there was a gap between both lateral incisors and canines, corresponding to the clinical situation after the first phase of en-bloc retraction. The model was then digitally divided into an anterior segment, consisting of the four incisors, and a posterior segment (Figure 1a,b).

In order to mount the posterior segment in the HOSEA test stand, a base part was designed using the software Autodesk Inventor 2021 (Autodesk Inc., San Rafael, CA, USA) (Figure 1b). To digitally determine the approximate center of resistance and the surface of the teeth in each direction for integration into HOSEA’s control software, four incisal roots were virtually modeled according to average root-to-crown ratios [26] using the software Autodesk Meshmixer Version 3.5 (Autodesk Inc., San Rafael, XA, USA). The virtual tooth models with roots were matched to the anterior segment in MeshLab [27], and the segment’s center of resistance was calculated. The final experimental model was additively manufactured from Grade 5 titanium powder (TiAl6V4) by selective laser melting (APWORKS Gmbh, Taufkirchen, Germany). The advantage of such a solid titanium model was its durability and stiffness, while a printed polymeric model is rather brittle and subject to extensive wear during multiple uses.

Self-ligating high torque brackets (0.022”, Damon Q, Ormco Corp, Pomona, CA, USA) were passively bonded to the titanium model using a 0.021” × 0.25” SS archwire, which was positioned vertically along the Andrews plane [28]. The starting position for the simulation was set to a 6.0 mm gap between the anterior and posterior segments.

### 2.2. Specimen

Four different groups of 10 archwires were examined with HOSEA in a simulated en-bloc retraction (Table 1). Two different mechanics for en-bloc retraction were investigated for two archwire dimensions each:

(1) Sliding mechanics with SS archwires (Forestadent GmbH, Pforzheim, Germany) and manually bent anterior torque in combination with elastic chains [5].
(2) Compound technique (modified sliding mechanics) with RTAs (Forestadent GmbH, Germany) out of NiTi and SS with prefabricated torque in combination with NiTi coil springs [5].

All archwires were adjusted to fit the maxillary model by the same experienced and trained clinician. Transversal adjustments were created using a template created with the archwire that was used for indirect bracket placement. The archwire shape was hence transversally adapted to the original passively fitting archwire. To achieve a passive fit, the archwires were flat and without vertical adjustments.

**Figure 2:**
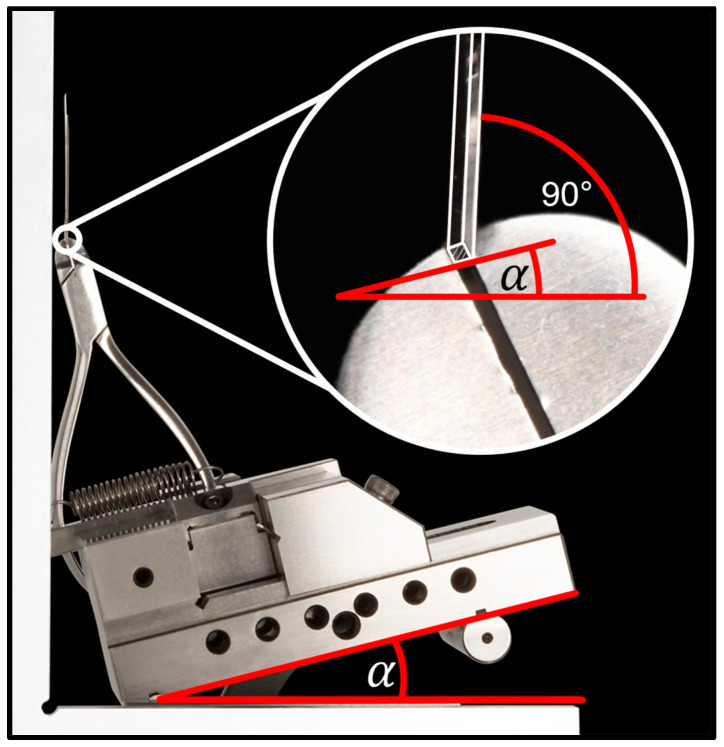
Torque measurement device. A tweed forceps was mounted on an adjustable parallel vice. After aligning the table using the clamped archwire parallel to 90° angle of the table plane, the anterior torque α (°) can be measured using a digital angle meter.

In addition to the transverse adjustments for archwire shape, twist bends were applied in the anterior region (lateral incisor to lateral incisor) in the SS groups. The amount of applied torque was controlled using a torque measurement device with a digital caliper with a resolution of ±0.01° (Figure 2). Values between 28° and 32° were considered acceptable with respect to the target value of 30° torque.

The RTAs did not require twist bends as they consisted of two lateral segments composed of stainless steel and a pretorqued anterior segment (30° torque) composed of Ni-Ti [16]. The segments are connected on both sides by an adjustable crimp connector with additional hooks for the attachment of NiTi springs. In this group, the crimp connection was positioned and fixed distally to the lateral incisor brackets, and the vertical hooks were bent to a length of 7 mm.

### 2.3. Biomechanical Simulation with HOSEA

**Figure 3:**
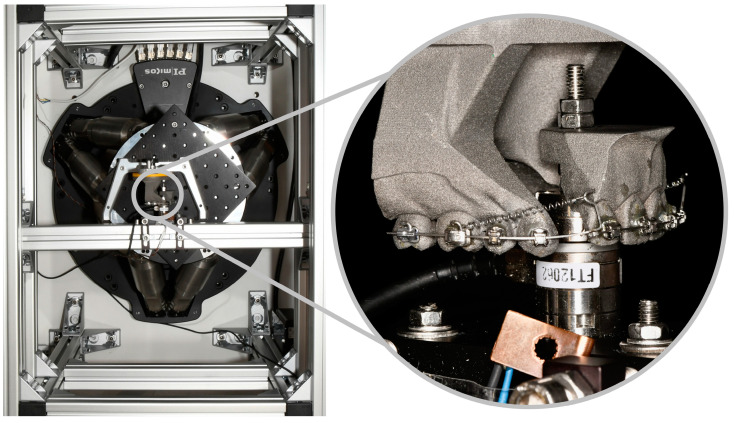
HOSEA’s temperature-controlled experimental chamber. Six hydraulic legs of the hexapod move the connected steward platform and the posterior part of the experimental model. The close-up depicts both model parts mounted within the experimental set-up. The anterior part is rigidly attached to a cross strut and force-torque sensor.

The posterior part of the experimental model was mounted to the Stewart platform via a base plate (SAM Praezisionstechnik GmbH, Munich, Germany). The Stewart platform is moved by six parallel electromotor actuators of the hexapod through control software (Figure 3). The retraction of the anterior segment is hence simulated by moving the posterior segment, while the anterior segment of the model is rigidly connected to a strut and a six-axis force-torque sensor (Nano17 SI-50-0.5, ATI Industrial Automation, Apex, NCUSA). For torsional moments, the sensor can measure up to 500 Nmm with a resolution of 1/16 Nmm in all three spatial planes. It detects a force range of 70 N in the axial direction and 50 N for two space vectors at a resolution of 1/80 N. As the specimen and the sensor show temperature-dependent behavior, the experiments were conducted at ϑ = (36 ± 1) °C in a temperature chamber equipped with a PID temperature controller (TOHO TM-105, TOHO electronics, Nishihashimoto, Japan).

Before the beginning of each experimental run, the preset hexapod's starting position was approached, with a distance of 6.0 mm between the distal edge of the lateral incisor and the mesial caninal approximal contact. Afterwards, the specimen and the retractive elements were mounted to the anterior and posterior model parts. The experimental cycle was started manually and was then autonomously conducted by the control software, depending on the measured forces and moments.

### 2.4. Statistical Analysis

IBM SPSS Statistics 26 (International Business Machines Corporation, Armonk, NY, USA) was used for statistical analysis. The mean maximal and mean initial force and moment values were analyzed with the help of descriptive statistics. Kolmogorov–Smirnov and Shapiro–Wilk tests were performed to test for normative distribution. Therefore, Kruskal–Wallis tests and post hoc tests with correction, according to Bonferroni, with a significance level of α = 0.05 were conducted to detect significant differences between the specimen groups (*p* < 0.05). Graphs were created using OriginPro 2022b (OriginLab Corporation, Northampton, MA, USA).

## 3. Results

**Figure 4:**
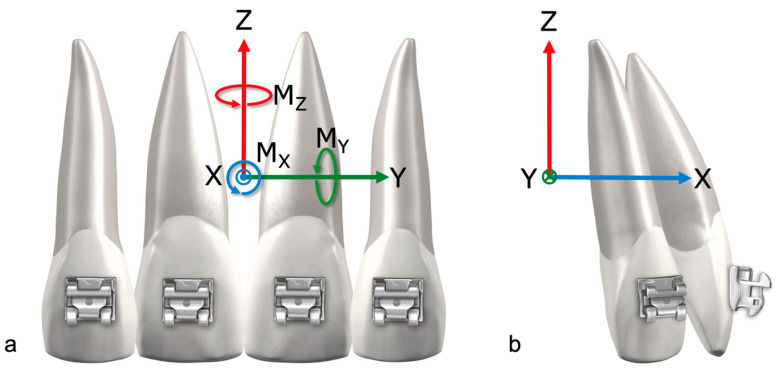
(**a**) Frontal view of four incisors with indication of the coordinate system, CoR (center of resistance) and CoF (center of force); (**b**) lateral view with indication of the coordinate system, CoR and CoF.

The measured forces and moments were transformed from the sensor position to the center of resistance as well as to the center of force of the anterior segment. The coordinate system describing the measurements has its origin in the center of resistance and describes the movements as a function of sign and direction (Figure 4a,b). Hence, for example, negative F_x_ values correspond to a retractive force and negative M_y_ values correspond to palatal root torque moments.

**Figure 5:**
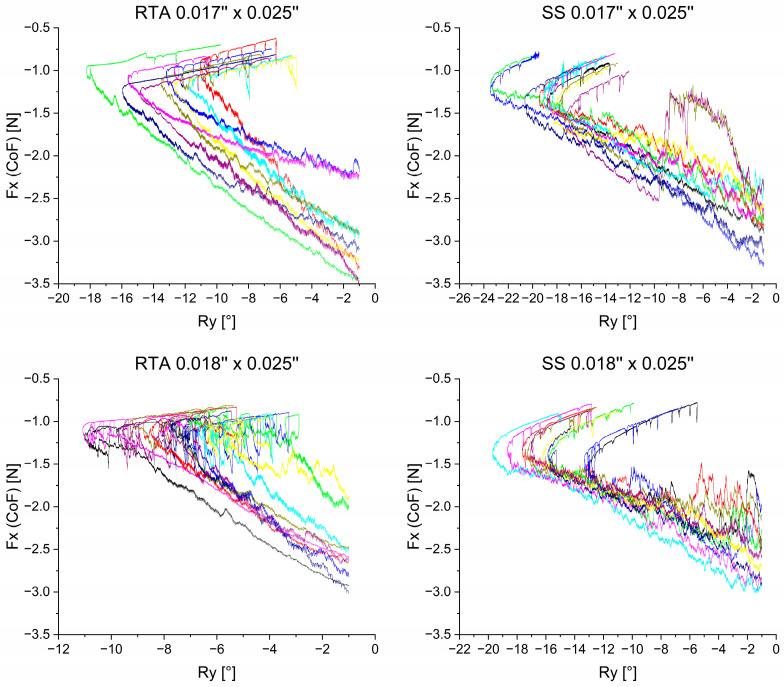
Retractive force F_x_ measured in the center of resistance plotted against the rotation angle of the anterior segment. The different colors represent different archwires. To account for initial irregularities, initial force values are depicted after −1° of rotation. While the ordinate is the same in all graphs, the abscissa, with the rotational angle R_y_ has a variable resolution.

The following results are displayed in combinatorial graphs to provide a differentiated graphical representation. The course of the measured forces is plotted for the four groups against the course of the rotation of the anterior segment (Figure 5). R_y_ was defined as a rotation angle around the y-axis and was chosen as abscissa because it provides relevant information about the tooth axes during retraction. The beginning of the measurements is at R_y_ = −1° to account for the initial irregularities of the force-controlled setup.

### 3.1. Retractive Force—F_x_

In all four groups, the amount of retractive force decreased throughout the simulated en-bloc retraction.

Initially, all four sample groups shared a reduction in R_y_ (Figure 5). This describes an initial palatal crown tipping movement of the anterior segment of teeth into the extraction space. Over the course of retraction, the direction of rotation changed, and an increase in R_y_ values was observed during the later stage of each experiment. Hence, the overall movement depicted by the graphs in Figure 5 is tipping, followed by an upright movement.

Two archwires in the 0.017” × 0.025” SS group showed irregular curves. During the experiment, both archwires led to an asymmetric retraction (Figure 5, olive green curve and plum purple curve). The 0.018” × 0.025” RTA explicitly exhibited a different behavior after reaching the maximum palatal tipping, characterized by a sequence of loop-like tipping and upright movements.

**Figure 6:**
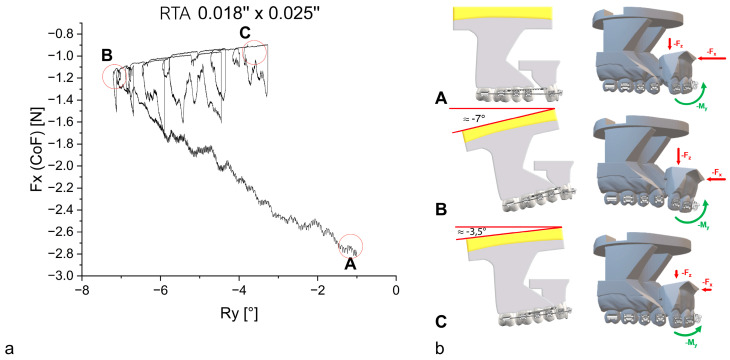
(**a**) The retractive force F_x_ is plotted against the rotation of the anterior segment of teeth for an 0.018” × 0.025” RTA. In order to reduce preliminary irregularities, initial force values are depicted after −1° of rotation. (**b**) Correlating graphics of the experimental model segments at the different time points (A, B, C) of en-bloc retraction. The rotating posterior segment is being moved by the hexapod device, while the anterior segment is statically fixed to the force-torque sensor, which represents an inversion of the in vivo process, showing force and moment vectors (F_x_, F_z_ and M_y_) on the anterior tooth segment. The lengths of the vectors represent the force and torque values schematically.

To analyze the irregular motion of en-bloc retraction in the 0.018” × 0.025” RTA group further, Figure 6 depicts a singular representative archwire of this group. At the starting position (A), a 6-mm gap between the segments is present. Subsequently, the anterior segment tipped palatally during retraction (A → B). Afterwards, the movement changed into an upright motion at around Ry= −7° (B). In the last phase, alternating tipping and upright movements were observed, characterized by loop-like patterns in the diagram (B → C). The final rotational position is reached after the retraction (C).

### 3.2. Rotational Moment—M_y_

**Figure 7:**
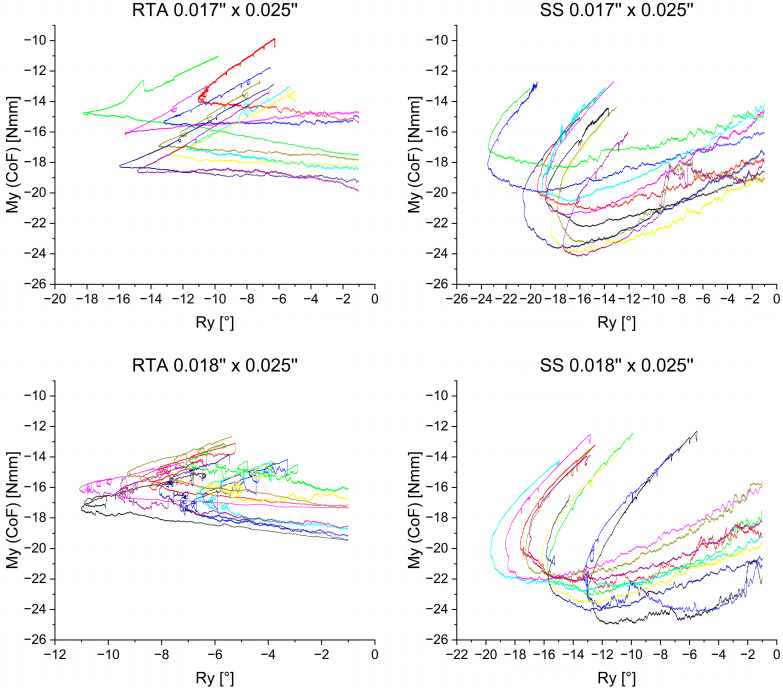
Rotational moment M_y_ measured in the center of resistance plotted against the rotation of the anterior segment. The different colors represent different archwires. To reduce preliminary irregularities, initial rotational moment values are depicted after −1° of rotation. While the ordinate is the same in all graphs, the abscissa with the rotational angle R_y_ has a variable resolution.

Figure 7 depicts the rotational moments M_y_ during en-bloc retraction. Negative M_y_ values correspond to a palatal root torque moment. The graphs differ primarily in two aspects: archwire material and archwire dimension. The archwire material had a significant influence on the M_y_ progression. The 0.017” × 0.025” RTA group showed mostly constant M_y_ values until the rotational direction changed and M_y_ magnitudes decreased. In the 0.018” × 0.025” RTA group, M_y_ amounts decreased even though R_y_ (°) values declined. In this group, a more considerable reduction in the M_y_ amount occurred with the rotational direction change. In this graph, the previously mentioned loop-like pattern could be observed, which indicates an alternating decrease and increase in torsional moment M_y_. The two RTA groups share the characteristic of an overall continuous M_y_ amount reduction over the course of retraction. This fact is further underlined in Table 2, as the corresponding R_y_ (°) of the maximum M_y_ values lie at −3.307° for 0.017” × 0.025” RTA’s and −1.410° for 0.018” × 0.025” RTA’s.

On the other hand, M_y_ magnitudes showed a different course in the SS groups (Figure 7). For these groups, M_y_ amounts increased until reaching their maximal amounts during the palatal tipping of the anterior segment and not at the beginning of the retraction. Specifically, with 0.017” × 0.025” SS archwires, the average minimum of −21.899 Nmm occurred at R_y_ = −16.573° of rotation, whereas the 0.018” × 0.025” SS group reached its M_y,max_ amount of 23.180 Nmm at R_y_ = −12.623° of rotation (Table 2).

### 3.3. Extrusive Force—F_z_

**Figure 8:**
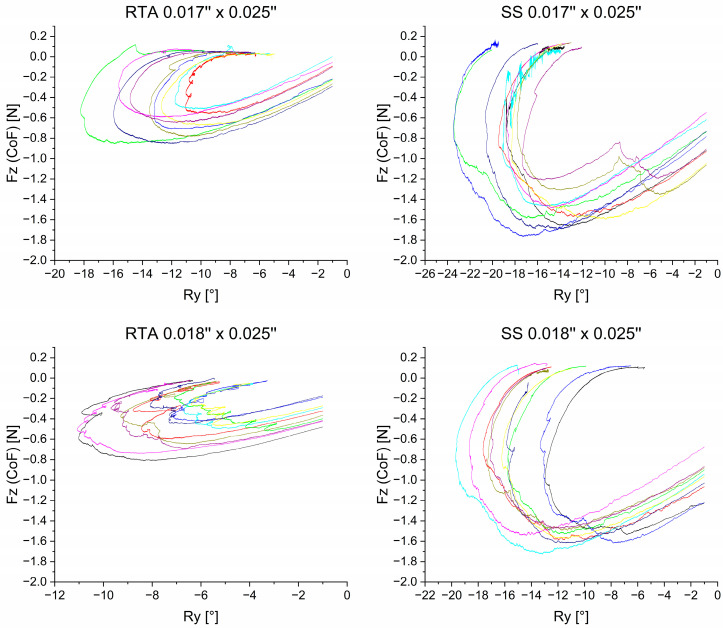
Extrusive force F_z_ measured in the center of resistance plotted against the rotation of the anterior segment. The different colors represent different archwires. To reduce preliminary irregularities, initial force values are depicted after −1° of rotation. While the ordinate is the same within the material groups (RTA and SS), the abscissa with the rotational angle Ry has a variable resolution.

Figure 8 illustrates the collateral extrusive force F_z_ occurring during retraction. In all four archwire groups, the amount of F_z_ increased, reached its maximum amount, and decreased again. The 0.017” × 0.025” and the 0.018” × 0.025” SS archwires reached the highest F_z_ amount at: F_z_= −1.540 N in the 0.017” × 0.025 “ dimension and F_z_ = −1.570 N in the 0.018” × 0.025”-dimension (Table 2). The maximal mean F_z_ values of the RTAs were less than half of the SS magnitudes. This fact was mirrored in the statistical analysis of the maximal values. Statistically significant differences occurred between the material RTA and SS but not between the individual archwire sizes.

### 3.4. Comparison between the Specimen Groups—Statistical Analysis

The maximum values of F_x,_ M_y_ and F_z_ with the corresponding R_y_ values are depicted in Table 2.

The maximum values of the retractive force F_x_ correlated to the initial rotational amounts, as R_y_ values are just below −1° of rotation. Even though no statistically significant differences of F_x_ magnitudes was detected between the groups, the absolute average F_x_ maximum values in the 0.017” × 0.025” archwire dimension were higher (F_x_ = −2.942 N for RTA 0.017” × 0.025”; F_x_ = −2.839 N for SS 0.017” × 0.025”) compared to the 0.018” × 0.025” specimen groups (F_x_ = −2.572 N for RTA 0.018” × 0.025”; F_x_ = −2.674 N for SS 0.018” × 0.025”). Statistically significant differences were observed for the palatal root torque moment M_y_ between RTA and SS groups. The RTA groups exhibited lower M_y_ magnitudes (M_y_ = −17.436 Nmm for RTA 0.017” × 0.025” and M_y_ = −18.099 Nmm for RTA 0.018” × 0.025”). The corresponding R_y_ was closer to −1° for the RTA groups in comparison to the SS groups. Further statistically significant differences were observed analyzing the maximum extrusive force magnitudes F_z_ comparing the RTA and SS groups. While the maximum F_z_ in the RTA groups reached magnitudes of F_z_ = −0.691 N for RTA 0.017” × 0.025”; F_z_ = −0.585 N for RTA 0.018” × 0.025”, the SS archwires expressed significantly higher values (F_z_ = −1.540 N for SS 0.017” × 0.025”; F_z_ = −1.570 N for SS 0.018” × 0.025”).

Focusing on the individual groups, differences between the maximum in rotation R_y,max_ were visible. The 0.017” × 0.025” SS archwire group showed the highest average rotational maximum of −19.717°, and therefore the highest amount of palatal tipping of the anterior segment. In contrast, the 0.018” × 0.025” RTA group exhibited the lowest average rotational maximum of −8.535°. In this study the rotational maxima of R_y_ showed great variation in values between the archwires within the groups (Figure 4, Figure 6 and Figure 7).

Upon reviewing the mean initial values for F_x_, M_y_ and F_z_, it was observed that only the extrusion force, F_z_, differed with statistical significance. The RTA groups exhibited lower initial values of F_z_ compared to the SS groups (Table 3).

## 4. Discussion

This study investigated the dynamic course of forces and moments during a simulated en-bloc retraction using the force-controlled biomechanical test stand HOSEA. Two different mechanics combined with two different archwire dimensions were investigated:

- SS archwires with dimensions 0.017” × 0.025” and 0.018” × 0.025” in combination with elastic chains and
- RTAs with dimensions 0.017” × 0.025” and 0.018” × 0.025” in combination with NiTi tension springs.

Regardless of the respective mechanics and archwire dimensions, all simulations showed a similar pattern. In the first phase, palatal tipping of the anterior teeth predominated during retraction. While the retractive forces (F_x_) continued to decrease, a reversal of the inclination changes and an uprighting of the anterior teeth was observed in the second stage (Figure 5). These results are in line with observations in literature about en-masse retraction [2,29]. The amount of palatal tipping was found to differ significantly between the investigated groups, with the type of mechanics having a greater effect than the modification of archwire dimensions within the same group. The SS 0.017” × 0.025” archwires exhibited the highest amount of palatal tipping (|R_y,max_| = 19.717°), in contrast to the 0.018” × 0.025” RTAs, which exhibited the least amount of palatal tipping (|R_y,max_| = 8.535°) (Table 2). Both RTA groups exhibited significantly less palatal tipping compared to the SS groups, most likely because the use of power-hooks in the RTA groups allowed for force application closer to the center of resistance [6,30], while the elastic chains applied in combination with the SS archwires delivered the retractive forces at the centre of force. In summary, the RTA 0.018” × 0.025” group allowed for the most bodily en-bloc retraction.

The retractive force F_x_ was found to vary depending on the archwire dimensions, although equal force magnitudes were applied for the respective groups. The 0.017” × 0.025” RTA and SS archwires showed higher maximal force magnitudes (|F_x,RTA_| = 2.942 N and |F_x,SS_| = 2.839 N) than the 0.018” × 0.025” groups (|F_x,RTA_| = 2.572 N and |F_x,SS_| = 2.674 N). This may be primarily attributed to increased friction with larger archwire dimensions [6,10,13], which seems to exceed the effect of the increased moment of inertia of the larger wire cross section. The mechanics used and force magnitudes applied were based on literature references [5]. However, the results of this study suggest that the applied retractive forces should be clinically adjusted to the archwire dimension to account for friction.

A large variation of Fx was also found at the beginning of the experiment within the different groups. This finding was more pronounced for the RTA mechanics, which used NiTi springs to generate the retractive forces. It is well known, that the force generated by identical NiTi springs may still vary based on two different aspects:

1. The test temperature. The thermocouple shown in Figure 3 measured the temperature close to the sample, but did not directly measure the exact NiTi wire temperature. It is well possible, that the time span between insertion of the archwire (sample setup) and the start of the experiment varied due to the manual application of the mechanics. Even though the experiment didn’t start before the set temperature of 36 °C was reached, the exact temperature in the NiTi wires may have been slightly different, thus causing variations in generated initial forces.
2. The over-straining during assembly. It makes a huge difference, if a NiTi spring is strained and immediately attached to the bracket or if it is overstrained and released a bit before attaching to the bracket. The latter procedure yields lower forces with same identical setup and sample. Even though the laboratory operators were made aware upon this effect, it cannot fully be excluded that some differences in attaching the springs may have occurred leading to the variability of the curves shown in Figure 5 for the RTA group.

Further research is going to take this eventuality into consideration by using calibrat-ed mechanical templates for the assembly and thermocouples with similar cross sections with and attached in closer proximity to the NiTi wires.

Taken together, significant differences in dynamically measured forces and moments were found comparing compound technique (RTA with NiTi-coil springs) to sliding me-chanics (SS with elastic chains) and regarding different archwire dimensions (0.017” × 0.025” vs. 0.018” × 0.025”. Hence, the null hypothesis can be rejected.

All investigated mechanics were used with anterior torque to control incisor inclination and prevent excessive palatal tipping during en-bloc retraction. The rotational moment M_y_, produced by the difference between torsional moments induced by the archwire in the brackets of the anterior segment and the pull-force of the spring or the chain, was found to vary in dependence on the archwire dimension and material. The reason is the constantly decreasing pull force from the chain as well as from the NiTi spring during retraction and the corresponding generated moments. Therefore, during the second phase of the process, the moment induced by the archwire in the bracket slot dominates and compensates more and more on the spring or chain generated moment, leading to the turnaround point found in all curves of Figure 7:

$$M_{y}={-M}_{spring/chain}+M_{archwire\;torque}$$

The different components of the above equation cannot be measured individually.

The RTAs exhibited maximum values of torsional moments close to the beginning of the simulated retraction and then showed a decreasing amount of M_y_. In contrast, the SS archwires showed an increase in the amount of M_y_ corresponding to an increase of palatal tipping of the anterior segment. The maximum torsional moments M_y_ for the 0.017” × 0.025” and 0.018” × 0.025” SS archwires exceeded recommended values between 5 Nmm and 20 Nmm with an |M_y,max_| = 21.899 Nmm and |M_y,max_| = 23.180, respectively [16,31]. Overload of the periodontal ligament can induce adverse effects like external apical root resorption (EARR) [18,32]. The application of lower torsional moments and reduced twist bends should therefore be considered when using SS archwires clinically. However, this could lead to more pronounced tipping of the anterior segment during retraction, necessitating further correction of the incisor inclination after space closure.

The material properties of the RTA and SS groups could account for the differing observations regarding the torsional moment M_y_ at identical archwire dimensions. RTAs are composed of an anterior pretorqued NiTi segment and two lateral stainless steel segments. The stress-strain curve of nickel-titanium with linear loading exhibits a so-called stress plateau, a phenomenon often referred to as superelasticity [33]. Within this stress plateau, the stress remains almost constant despite the increasing strain, caused by a martensitic phase transformation in the NiTi crystal structure from the austenite to the martensite. In the context of the present investigation, palatal tipping of the anterior segment results in increased strain. However, it is apparent that in the RTA groups, M_y_ values do not change significantly (Figure 7). In comparison, the SS archwires, which do not exhibit superelasticity, showed a linear increase of M_y_ corresponding to the amount of tipping (Figure 7, Table 2). Thus, overload of the periodontal ligament may occur due to pronounced tipping of the anterior segment and increase of M_y_, even if reduced twist bends are used with SS archwires.

The use of NiTi archwires has mostly been proposed for the leveling phase of fixed orthodontic treatment, given their low Young’s modulus and superelastic behavior especially during unloading. The results of this study imply that NiTi components, integral to RTAs, are beneficial in preventing overactivation during complex tooth movements during later treatment stages, such as en-bloc retraction while still yielding similar results in overall movement of the anterior segment.

**Figure 9:**
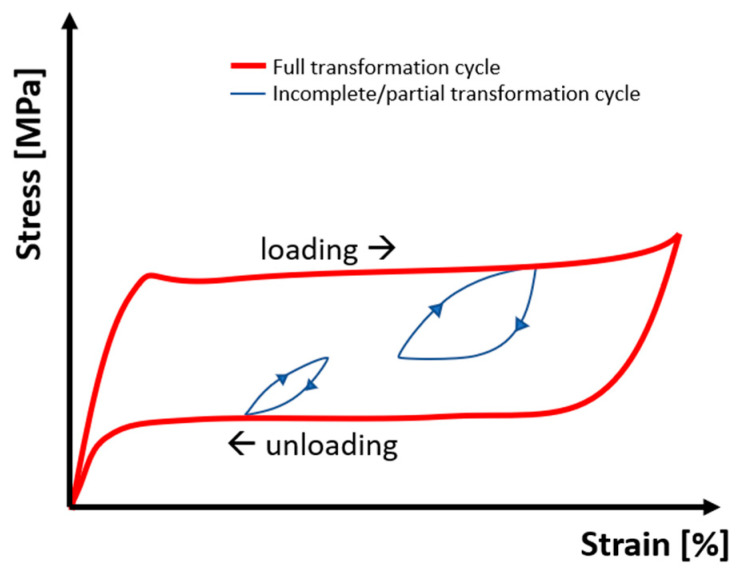
Schematic depiction of the stress-strain behavior of superelastic NiTi alloys in case of interrupted loading or unloading cycles [35].

The material properties may also explain another observation of the simulations. In addition to the observed pattern of tipping predominating in the first phase of en-bloc retraction and uprighting in the second phase, the moment curves showed multiple smaller deflections with smaller tipping-uprighting loops (see Supplementary Materials, Video S1). This is consistent with the literature and biomechanical understanding, as a bodily movement is described as the result of alternating tipping and uprighting movements [5]. However, these deflections were particularly evident and pronounced in the RTA groups, especially for the 0.018” × 0.025” RTAs and are caused by the specific behavior of NiTi alloys in case of incomplete and interrupted loading-unloading cycles [34] (Figure 9).

In addition to retractive forces F_x_ and torsional moments M_y_, collateral forces and moments were measured, in particular an extrusive component F_z_ (Figure 8, Table 2). The occurrence of an extrusive force component during retraction has been described in literature [21,36]. For this reason, it is recommended to apply compensatory bends during or after retraction [20,36]. In this study the aim was to focus on the forces and moments during en-bloc retraction, thus compensatory bends were not applied. The force magnitudes F_z_ differed between the archwire dimensions and archwire groups and would be of clinical relevance in all groups investigated. SS groups showed a statistically significant greater extrusive force magnitudes for both archwire dimensions (F_z, SS17×25_ = −1.540 N; F_z, SS18×25_ = −1.570 N) compared to RTA groups (F_z, RTA17×25_ = −0.691 N; 0.018” × 0.025”, F_z, RTA18×25_ = −0.585 N). The reasons for this observation may be the differing amounts of palatal tipping, which was higher in the SS groups, and the use of power-hooks in the RTA groups, allowing for force application closer to the center of resistance of the incisors.

Biomechanical test stands can only reflect clinical processes to a certain extent and have inherent limitations [23,37]. First of all, the periodontal ligament could not be adequately accounted for within the test setup. Thus, the center of resistance and the force-control feedback parameters were determined statically. Furthermore, a common center of resistance was assumed for the four incisors, although this is not exactly appropriate as demonstrated in finite element simulations [38]. Clinically, the center of resistance was found to be dynamic during orthodontic tooth movements [39]. In addition, the experimental model used in this study consisted of two solid segments, without allowing to account for individual tooth mobility. However, even if the teeth are combined to a block using figure-8 steel ligatures, individual tooth mobility remains [36]. The setting did not include saliva, which can influence frictional behavior and thus affect the measured values [40]. Another limitation is the variability of the manual adjustment of the archwires. Although the adjustments were performed by an experienced practitioner and verified using a measurement apparatus and a template, variability in the measured values was observed. This is apparent in the graphs, as well as in large standard deviations. The variability reflects the clinical application of archwire bending, which is associated with a certain degree of inaccuracy, which can be assumed to be even more pronounced in routine clinical practice. Finally, two distinct mechanical approaches were compared, which differ in terms of the retractive elements and the point of force application. Although the chosen mechanics are based on literature recommendations and reflect their clinical application, these aspects have an influence on the measured forces and moments which was not quantified. Thus, the results provide insight in particular with respect to the initial and maximum forces and moments regarding the specific mechanical approaches. In the clinical setting, elastic chains are commonly applied over several weeks, during which a force decay ranging from 25–65% is to be expected [7]. Therefore, the clinical procedure might differ from the simulated course of en-bloc retraction which usually ranges from two to three hours.

Within the limitations of the experimental setup, HOSEA allows for the force-controlled investigation of orthodontic appliances and the quantification of forces and moments during simulated clinical procedures. In contrast to comparable biomechanical studies, the measurements are independent of predefined measurement points, distances or angles that may not represent the clinical treatment process [41,42].

## 5. Conclusions

The biomechanical simulations showed that en-bloc retraction is characterized by a series of alternating tipping and uprighting movements. RTAs showed less palatal tipping of the incisors during retraction compared to SS archwires. SS archwires exhibited excessive maximum moments above 20 Nmm and therefore should not be used clinically in the form studied. The use of SS archwires in other configurations may also lead to periodontal overloading, as the strain is increased by the palatal tipping of the incisors during en-bloc retraction. The incorporation of NiTi components in RTAs has been shown to be effective in preventing overactivation in this respect.

Among the different mechanics compared, the 0.018” × 0.025” RTA showed the most bodily en-bloc retraction with the least amount of palatal tipping.

## Figures and Tables

**Table 1:** Specimen groups investigated, their material composition, the amount of anterior torque, the archwire dimensions and the retractive elements used.

Archwire Group	Material Identification	Anterior Torque α (°)	Sample Size	Archwire Size Anterior Segment	Archwire Size Posterior Segments	Retractive Element
SS	X10CrNi 18-8	28–32	10	0.017” × 0.025”	0.017” × 0.025”	Elastic chain
SS	X10CrNi 18-8	28–32	10	0.018” × 0.025”	0.018” × 0.025”	Elastic chain
RTA	X5CrNi18-10 Nickel Titanium	30	10	0.017” × 0.025”	0.017” × 0.022”	Nitinol spring
RTA	X5CrNi18-10 Nickel Titanium	30	10	0.018” × 0.025”	0.018” × 0.022”	Nitinol spring

**Table 2:** Mean maximum amounts and 95% confidence interval (CI) of F_x_, M_y_ and F_y_ with the corresponding average degree of rotation and the rotational maxima R_y max_ (°). Statistically significant differences between groups (a,b,c,d) in F_x_, M_y_ and F_y_ values are indicated by the same superscript letters (a,b,c,d). Kruskal–Wallis tests with post hoc correction according to Bonferroni were performed to determine these differences, with a significance level set to *p* < 0.05.

Sample Group		F_x,max_[N]	R_y,corr_ [°]	M_y,max_ [Nmm]		R_y,corr_ [°]	F_z,max_ [N]		R_y,corr_ [°]	R_y max_ [°]	
(a) RTA 0.017” × 0.025”	Mean (SD)	−2.942 (0.497)	−1.312 (0.340)	−17.436 (1.645)	^c,d^	−3.307 (4.573)	−0.691 (0.117)	^c,d^	−11.716 (1.776)	−14.008 (2.157)	^b,c^
	CI [95]	[−3.298; −2.586]	[−1.555; −1.069]	[−18.613; −16.259]		[−6.578; −0.036]	[−0.774; −0.607]		[−12.986; −10.445]	[−15.551; −12.465]	
(b) RTA 0.018” × 0.025”	Mean (SD)	−2.572 (0.350)	−1.089 (0.114)	−18.099 (1.154)	^c,d^	1.410 (0.785	−0.585 (0.134)	^c,d^	−6.370 (1.528)	−8.535 (1.718)	^c,d^
	CI [95]	[−2.823; −2.322]	[−1.170; −1.008]	[−18.925; −17.274]		[−1.972; −0.849]	[−0.681; −0.489]		[−7.464; −5.277]	[−9.764; −7.305]	
(c) SS 0.017” × 0.025”	Mean (SD)	−2.839 (0.239)	−1.316 (0.456)	−21.899 (1.897)	^a,b^	−16.573 (1.371)	−1.540 (0.166)	^a,b^	−13.492 (3.641)	−19.717 (2.164)	^a,b^
	CI [95]	[−3.009; −2.668]	[−1.642; −0.990]	[−23.256; −20.541]		[−17.553; −15.592]	[−1.658; −1.421]		[−16.097; −10.888]	[−21.265; −18.170]	
(d) SS 0.018” × 0.025”	Mean (SD)	−2.674 (0.247)	−1.551 (0.831)	−23.180 (1.015)	^a,b^	−12.623 (2.662)	[−1.570 (0.073)	^a,b^	−11.168 (2.266)	−16.452 (2.126)	^b^
	CI [95]	[−2.850; −2.497]	[−2.145; −0.957]	[−23.906; −22.454]		[−14.527; −10.718]	[−1.622; −1.518]		[−12.789; −9.547]	[−17.972; −14.931]	

**Table 3:** Mean initial values and 95% confidence interval (CI) of F_x_, M_y_ and F_z_ at a rotational degree of >1°. Significant differences between groups (a,b,c,d) in F_x_, M_y_ and F_z_ values are indicated by the same superscript letters (a,b,c,d). Kruskall–Wallis tests with post hoc correction according to Bonferroni were conducted. The *p*-value was set at *p* < 0.05.

Sample Group		F_x_ [N]	M_y_ [Nmm]	F_z_ [N]	
(a) RTA 0.017” × 0.025”	Mean (SD)	−2.891 (0.513)	−17.126 (1.904)	−0.169 (0.099)	^c^^,^^d^
	CI [95]	[−3.258; −2.525]	[−18.488; −15.764]	[−0.240; −0.098]	
(b) RTA 0.018” × 0.025”	Mean (SD)	−2.549 (0.358)	−17.996 (1.212)	−0.332 (0.104)	^c^^,^^d^
	CI [95]	[−2.805; −2.293]	[−18.863; −17.129]	[−0.407; −0.258]	
(c) SS 0.017” × 0.025”	Mean (SD)	−2.720 (0.304)	−16.885 (1.898)	−0.827 (0.174)	^a^^,^^b^
	CI [95]	[−2.937; −2.502]	[−18.243; −15.527]	[−0.951; −0.703]	
(d) SS 0.018” × 0.025”	Mean (SD)	−2.521 (0.316)	−18.920 (2.116)	−0.976 (0.167)	^a^^,^^b^
	CI [95]	[−2.747; −2.295]	[−20.434; −17.406]	[−1.095; −0.856]	

## Data Availability

The complete datasets are available from the corresponding author upon reasonable request.

## Supplementary Materials

The following supporting information can be downloaded at https://www.mdpi.com/article/10.3390/bioengineering11020153/s1, Video S1: RTA.
